# Effects of different water conditions on the biomass, root morphology and aerenchyma formation in bermudagrass (*Cynodon dactylon* (L.) Pers)

**DOI:** 10.1186/s12870-022-03653-2

**Published:** 2022-05-30

**Authors:** Zhongxun Yuan, Xilu Ni, Chunhua Chen, Songlin Zhang, Xuemei Chen, Zhihua Yang, Changxiao Li

**Affiliations:** 1 Key Laboratory of Eco-Environments in the Three Gorges Reservoir Region (Ministry of Education), Chongqing Key Laboratory of Plant Resource Conservation and Germplasm Innovation, College of Life Sciences, Southwest University, Chongqing, 400715 China; 2 State Cultivation Base of Eco-Agriculture for Southwest Mountainous Land, Southwest University, Chongqing, 400715 China; 3 Breeding Base for State Key Laboratory of Land Degradation and Ecological Restoration of North-Western China, Key Lab for Restoration and Reconstruction of Degraded Ecosystem in North-Western China (Ministry of Education), Ningxia University, Yinchuan, 750021 China; 4 Key Laboratory of Reservoir Aquatic Environment, Chongqing Institute of Green and Intelligent Technology, Chinese Academy of Sciences, Chongqing, 400714 China

**Keywords:** Aerenchyma, Bermudagrass, Root cavity rate, Root growth, Submergence

## Abstract

**Background:**

The bermudagrass (*Cynodon dactylon* (L.) Pers) roots responded differently in terms of morphological and anatomical characteristics under diverse submergence conditions, and they developed aerenchyma under non-flooding condition. In order to understand these mechanisms, bermudagrass cuttings were used as experimental material to examine their biomass, root morphology, and aerenchyma formation under three different water treatments, including control (CK), shallow submergence (SS), and deep submergence (DS).

**Results:**

The total root length, root volume, root surface area, and biomass of bermudagrass were largest in CK, followed by SS and DS. However, the average root diameter was greater in each of DS and SS than that in CK. Root aerenchyma formation was observed in CK, and submergence boosted the aerenchyma formation and the root cavity rate. Furthermore, our study found that the process of aerenchyma formation began with the increase of cell volume and cell separation to form a narrow space, and these cells gradually died to form matured aerenchyma cavity, which belongs to schizo-lysigenous aerenchyma. Meanwhile, typical biomarkers of programmed cell death were also observed.

**Conclusion:**

Overall, these results suggested that submergence inhibited the accumulation of biomass and root growth, but facilitated aerenchyma formation by increasing root diameter.

## Background

Submergence stress is one of the main abiotic stresses affecting plant survival and growth. Submergence occurs in most of the world every year at varying degrees and times [1], including the hydro-fluctuation belt formed by reservoir construction. The frequency of occurrence of submergence stress in riparian zones of the reservoirs is higher, and the negative impact is more severe by increased flooding. The Three Gorges Dam (TGD), the largest dam in China built on the upper Yangtze river, was constructed for flood control, hydropower generation and navigation [2]. After the initial formal impoundment in 2008, the annual water level of the reservoir fluctuated between the lowest water level of 145 m a.s.l. in summer and the highest water level of 175 m a.s.l. in winter [3]. Thus, a hydro-fluctuation belt was formed with a length of over 2000 km, a vertical drop of 30 m and an area of 400 km^2^, which transformed the original terrestrial ecosystem into a seasonal wetland ecosystem. Contrary to nature, the newly formed hydro-fluctuation pattern has changed the hydrological characteristics of the TGD reservoir to anti-seasonal and prolonged submergence, which completely altered the habitat of natural vegetation [4]. Many native plants have thus gradually died off due to extreme habitat changes [5]. This has led to a series of ecological and environmental problems such as soil erosion, habitat loss, biodiversity decline, and environmental pollution in the reservoir area [6].

Submergence damage on plants is mainly due to the limited oxygen availability, because the diffusion rate of gas in water is 10,000 times slower than that in air [7, 8]. The solubility of oxygen in the water is relatively low, as 1 L of air contains 33 times more oxygen than 1 L of water at 20℃ at sea level [9]. Coupled with the root system's own energy consumption, the consumption of soil animals and microorganisms can further lead to a lack of oxygen in plant roots [10]. Root oxygen deficiency limits the submergence plant’s aerobic respiration and energy provision (such as adenosine triphosphate (ATP)) necessary to sustain the life activities. Under submergence conditions, the plant’s mineral element absorption capacity is usually lowered, while there is an increase in plant’s metabolic toxin element produced by anaerobic metabolism such as Mn^2+^, Fe^2+^, and S^2−^, and the like [11, 12]. Meanwhile, gas diffusion is blocked under the water, leading to the accumulation of a large number of volatile compounds, such as carbon dioxide, methane, and ethylene. Compared with unsubmerged plant tissues, ethylene in submerged roots can reach a high level of 20 times (1 μl/L) in one hour under submergence conditions [13], and the concentration of nitric oxide and reactive oxygen species (ROS) can also be changed [14]. A large amount of ROS can cause plant metabolism inactivation, cell death, photosynthetic rate decline, and so on, and have obvious toxic effects on plants [15]. Thus, submergence can lead to energy and toxicity crises in plants, which seriously affects root growth. Stagnation of growth and development and loss of biomass eventually leads to plant death [16]. Therefore, it is of great importance for plants to survive in submergence conditions through transporting oxygen to the root system to maintain aerobic respiration and diffusing internal gas to the outside.

Aerenchyma consists of longitudinally interconnected gas spaces that allow rapid transport of gases (e.g. oxygen, carbon dioxide, ethylene and methane) between and within the aboveground part and root [17–20]. For example, aerenchyma can transport carbon dioxide produced by root respiration [21] or from waterlogged soil to the leaves’ intercellular space to facilitate the photosynthesis. Furthermore, aerenchyma can also provide a channel for the movement of carbon dioxide from the rhizosphere to the aboveground part when being completely submerged [22]. At the same time, the removal of cells during the aerenchyma formation can reduce energy consumption and nutrient requirements. Very apparently, the formation of aerenchyma enhances the plant’s tolerance to submergence, which is the main physiological and morphological response of plants to submergence [23–26].

Studies have found that the mechanism of aerenchyma formation mainly includes the following two types: one is schizogenous, which is the result of cell separation in the inner cortex and cell expansion in the peripheral cortex; the other is lysigenous, where the internal or external stimuli cause cortical cells to selectively die through programmed cell death (PCD) [18, 27, 28]. Although the two processes are different, their key roles are both to provide space for the storage and transport of gas inside and maintain the physiological metabolism of plant roots [18]. In fact, there is no absolute distinction between schizogenous aerenchyma and lysigenous aerenchyma. Schizo-lysigenous aerenchyma is also present in some plants, for which plant cells separate to produce gas space, followed by some cells’ death [20, 25, 27, 29]. Nevertheless, some of the cells in the lysogenous process die to form a cavity while others are used as a scaffold to connect cavities without death. The radial scaffolds formed by these radial cell units and cell wall residues are important for the structural integrity of the root and for both symplastic transport of ionic nutrients and apoplastic transport of water [30].

Bermudagrass (*Cynodon dactylon* (L.) Pers) is a native species growing in the hydro-fluctuation belt of the Three Gorges Reservoir (TGR). This species has a strong tolerance of submergence and can withstand deep and continuous submergence stress for half a year [31]. Previous studies have documented the physiological and biochemical responses of bermudagrass to submergence [11, 32, 33]. But, little information is available on the mechanism of root morphological and anatomical (aerenchyma) response of bermudagrass to submergence. Although a former study reported that bermudagrass root could form lysigenous aerenchyma under non-flooding condition [34], the mechanism and process of aerenchyma formation in bermudagrass root under such a treatment were not scientifically and accurately elucidated [34]. Meanwhile, the former study did not explore how the root morphology and anatomy (aerenchyma) in bermudagrass changes under different submergence conditions. To fill this knowledge gap, we studied the root morphological characteristics, aerenchyma changes and plant biomass in bermudagrass under different submergence conditions, especially the temporal changes of aerenchyma formation, and further investigated whether PCD occurred during aerenchyma formation, so as to scientifically and accurately determine the manner of aerenchyma formation in bermudagrass. Thus, we hypothesize that: 1) Bermudagrass responds to submergence conditions by changing its root morphology and increasing aerenchyma formation; 2) PCD occurs during the formation of aerenchyma in bermudagrass root, which belongs to schizo-lysigenous process. The results obtained in the study are of great significance to reveal the adaptive mechanism of bermudagrass roots to different submergence and the manner of aerenchyma formation under non-flooding condition.

## Results

### Biomass

The effects of submergence treatment, sampling time, and their interactions on the shoot biomass, root biomass, total biomass, and ratio of shoot to root in biomass (RSR) of bermudagrass reached a highly significant level (*P* < 0.001) (Table 1). Across the whole experimental periods, the shoot biomass, root biomass, and total biomass showed a continuous increase in CK, in contrast to a first decrease then increase in SS and a continuous decline in DS (Table 2). Furthermore, at 60 days post treatment, there was no significant difference in shoot biomass and total biomass between CK and SS (all *P* > 0.05), respectively. Meanwhile, the shoot biomass and total biomass in DS were significantly lower than that in CK and SS (all *P* < 0.05), respectively. In addition, significant difference was detected in root biomass among the three treatments at 60 days post treatment (all *P* < 0.05). The root biomass of CK was 2.48 and 70.1 times that of SS and DS, respectively.

**Table 1:** Results of Two-way ANOVA for biomass, root growth properties of bermudagrass

Variables	Sampling time		Submergence treatment		Sampling time x Submergence treatment	
	***F*-value**†	***P*-value**	***F*-value**	***P*-value**	***F*-value**	***P*-value**
Shoot biomass (mg)	21.099	< 0.001	71.715	< 0.001	14.22	< 0.001
Root biomass (mg)	80.6	< 0.001	317.122	< 0.001	50.607	< 0.001
Total biomass (mg)	25.057	< 0.001	86.089	< 0.001	16.532	< 0.001
Shoot/ root biomass (mg/mg)	3.014	0.008	153.64	< 0.001	23.508	< 0.001
Total root length (cm)	206.492	< 0.001	1552.552	< 0.001	158.154	< 0.001
Root volume (cm^3^)	95.94	< 0.001	959.055	< 0.001	105.75	< 0.001
Root surface area (cm^2^)	193.069	< 0.001	1635.189	< 0.001	170.199	< 0.001
Root surface area/ Root volume (cm^2^/cm^3^)	73.078	< 0.001	93.632	< 0.001	11.929	< 0.001
Average root diameter (mm)	69.536	< 0.001	75.699	< 0.001	7.097	< 0.001

†The *F*-value is the value of the F-test

**Table 2:** Biomass (dry weight) of bermudagrass at different sampling times under different treatments

	**Shoot biomass (mg)**			**Root biomass (mg)**			**Total biomass (mg)**		
Time	CK	SS	DS	CK	SS	DS	CK	SS	DS
1 d	106 ± 8.59Aa^†^	110 ± 12.7Abc	102 ± 9.18Ad	3.51 ± 0.330Aa	3.49 ± 0.330Aab	3.74 ± 0.420Ad	109 ± 8.81Aa	114 ± 13.0Abc	106 ± 9.49Ae
5 d	112 ± 5.88Aa	107 ± 13.1Abc	92.3 ± 8.01Acd	4.82 ± 0.300Bb	3.36 ± 0.310Aa	3.41 ± 0.200Ad	117 ± 5.87Aa	111 ± 13.3Abc	95.7 ± 8.08Ade
10 d	124 ± 9.00Ba	86.3 ± 8.69Aab	80.0 ± 5.35Abc	7.32 ± 0.470Bc	2.86 ± 0.280Aa	2.29 ± 0.250Ac	131 ± 9.20Ba	89.2 ± 8.85Aab	82.3 ± 5.49Acd
15 d	129 ± 13.4Bab	69.1 ± 5.43Aa	77.8 ± 9.23Abc	12.6 ± 1.13Bd	2.44 ± 0.360Aa	2.10 ± 0.310Ac	142 ± 14.2Bab	71.5 ± 5.70Aa	79.9 ± 9.40Abd
20 d	167 ± 13.7Bb	140 ± 13.0Bc	65.7 ± 5.27Ab	14.2 ± 1.42Cd	2.86 ± 0.300Ba	1.82 ± 0.180Abc	182 ± 14.8Cb	143 ± 13.3Bc	67.5 ± 5.40Abc
30 d	337 ± 36.2Cc	142 ± 12.9Bc	61.3 ± 5.72Ab	30.9 ± 2.40Ce	4.37 ± 0.230Bb	1.20 ± 0.100Ab	368 ± 38.2Cc	146 ± 13.1Bc	62.5 ± 5.78Ab
60 d	316 ± 48.1Bc	273 ± 31.4Bd	38.6 ± 3.36Aa	39.9 ± 3.37Ce	16.1 ± 1.89Bc	0.570 ± 0.0600Aa	356 ± 48.5Bc	289 ± 32.4Bd	39.2 ± 3.35Aa

†The data in the table are the mean ± standard error (SE) (*n* = 10). Uppercase letters (A-C) indicate significant difference between different treatments at the same sampling time in each indicator (*P* < 0.05), and lowercase letters (a-e) indicate significant difference between different sampling time under the same treatment in each indicator (*P* < 0.05)

*CK* Control, *SS* Shallow submergence, *DS* Deep submergence

**Fig. 1:**
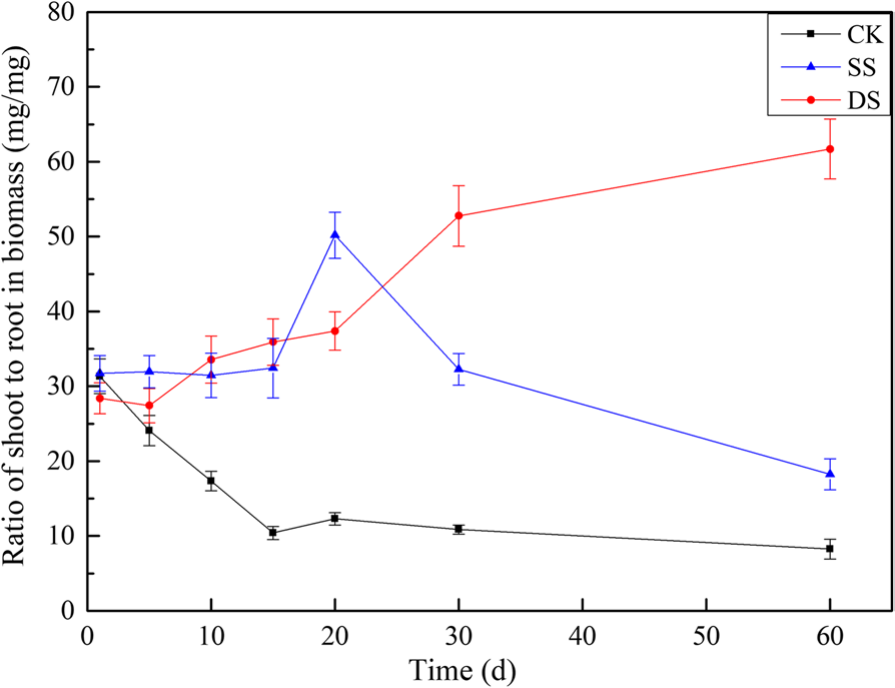
Ratio of shoot to root in biomass (RSR) of bermudagrass under control (CK), shallow submergence (SS), and deep submergence (DS) treatment. Values are mean ± standard error (SE) (*n* = 10)

The RSR in CK significantly decreased (*P* < 0.05) when the treatment duration was less than 15 days and showed a trend of decreasing at 15 days post treatment (Fig. 1). In contrast, the RSR in SS showed a relatively stable trend before 15 days and up to maximum at 20 days then significantly decreasing (*P* < 0.05). However, the RSR in DS showed continuously increasing. At 60 days post treatment, there was a significant difference in RSR among the three treatments (all *P* < 0.05). The RSR in DS was 3.38 and 7.49 times that in SS and CK, respectively. Furthermore, the RSR in SS was 2.21 times that in CK (*P* < 0.05).

### Root growth parameters

**Fig. 2:**
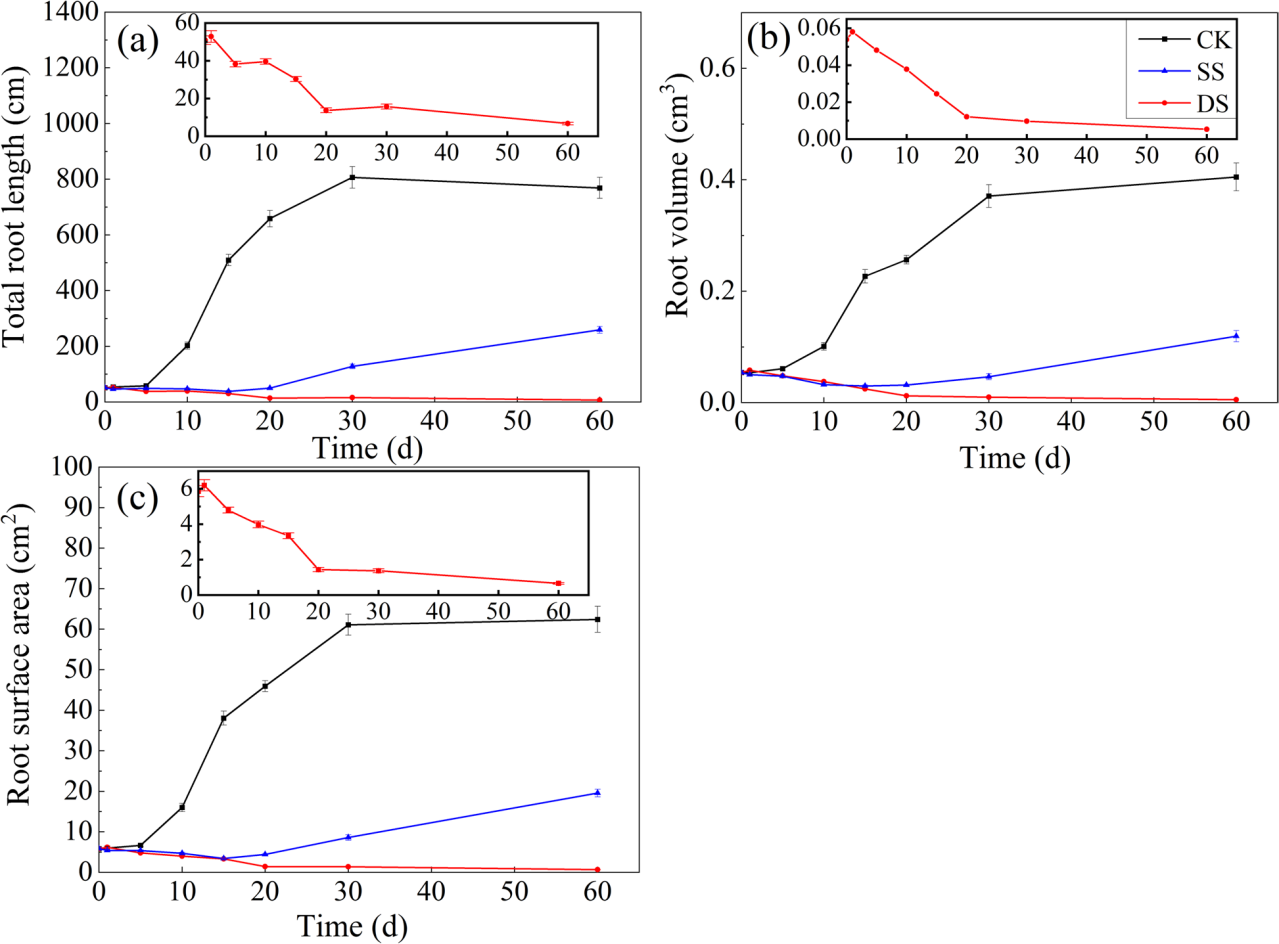
Total length (**a**), Root volume (**b**), and Root surface area (**c**) of bermudagrass under control (CK), shallow submergence (SS), deep submergence (DS) treatment. Values are mean ± standard error (SE) (*n* = 10). The inserted figure was to better show the mean ± standard error (SE) of DS

The effects of submergence treatment, sampling time, and their interactions on total root length, root volume, root surface area, surface area per unit volume of root, and average root diameter of bermudagrass reached a highly significant level (*P* < 0.001) (Table 1). At all sampling times, the total root length, root volume, and root surface area of bermudagrass were much larger in CK than in each of SS and DS (Fig. 2). Along with the experiment progressing, the total root length, root volume, and root surface area of bermudagrass showed continuously increasing in CK, in contrast to a trend of decreasing before 15 days and then significantly increasing (*P* < 0.05) in SS, and a continuous decline in DS. Moreover, at 60 days post treatment, there was a significant difference in the total root length, root volume, and root surface area among the three treatments (all *P* < 0.001). The total root length, root volume, and root surface area of bermudagrass in CK were 2.97, 3.42, and 3.19 times that in SS and 115, 82.0, and 94.6 times that in DS, respectively.

**Fig. 3:**
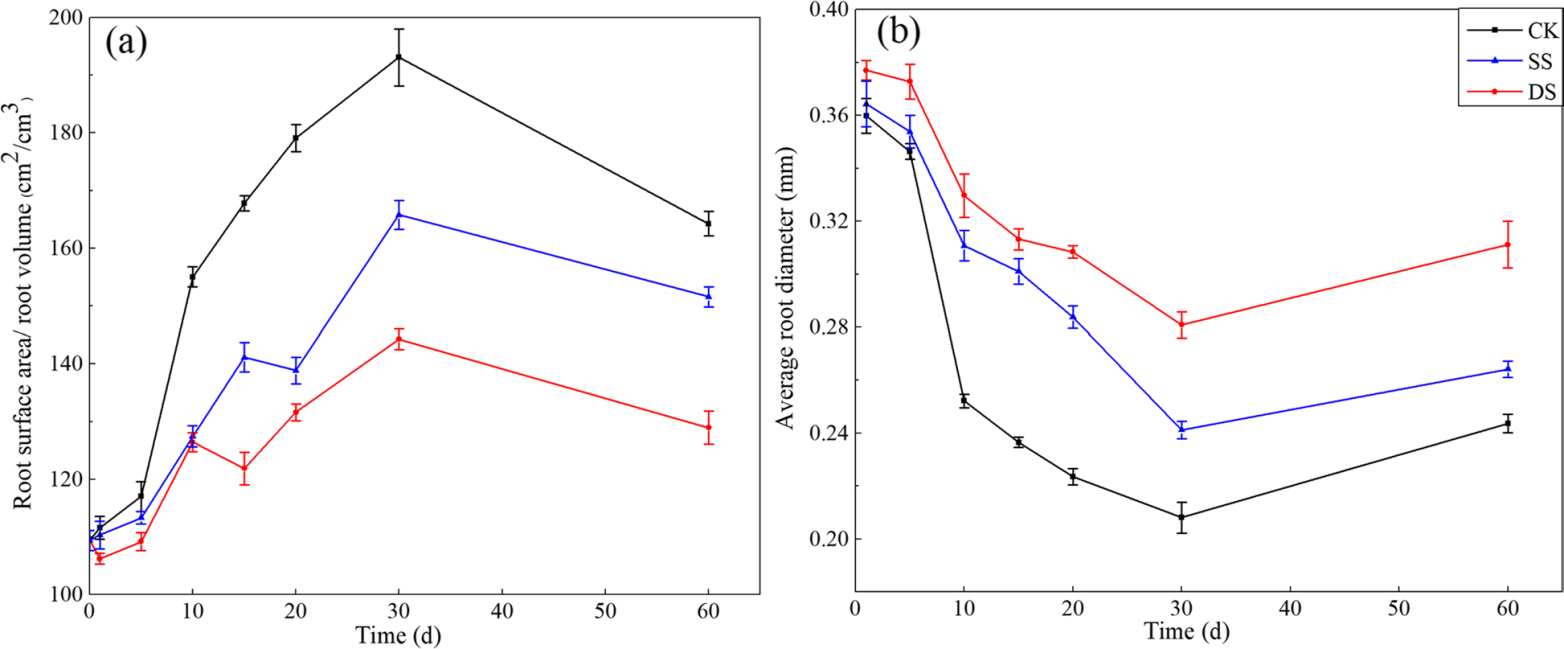
Surface area per unit volume of root (**a**), Average root diameter (**b**) of bermudagrass under control (CK), shallow submergence (SS), and deep submergence (DS) treatment. Values are mean ± standard error (SE) (*n* = 10)

At all sampling times, the surface area per unit volume of root was largest in CK, followed by SS and DS successively. For the whole experimental period, the surface area per unit volume of root firstly increased and then decreased, with a maximal value at 30 days (Fig. 3a). As expected, the average root diameter was opposite to the change of surface area per unit volume of root (Fig. 3b). There was a significant difference in the surface area per unit volume of root and average root diameter, respectively, among the three treatments at 60 days post treatment (all *P* < 0.001). In addition, at 60 days post treatment, the surface area per unit volume of root in CK was 1.08 and 1.27 times that in SS and DS, respectively; in contrast, the average root diameter in CK was about 92% and 77% of that in SS and DS, respectively.

### Changes of aerenchyma and root cavity rate

**Fig. 4:**
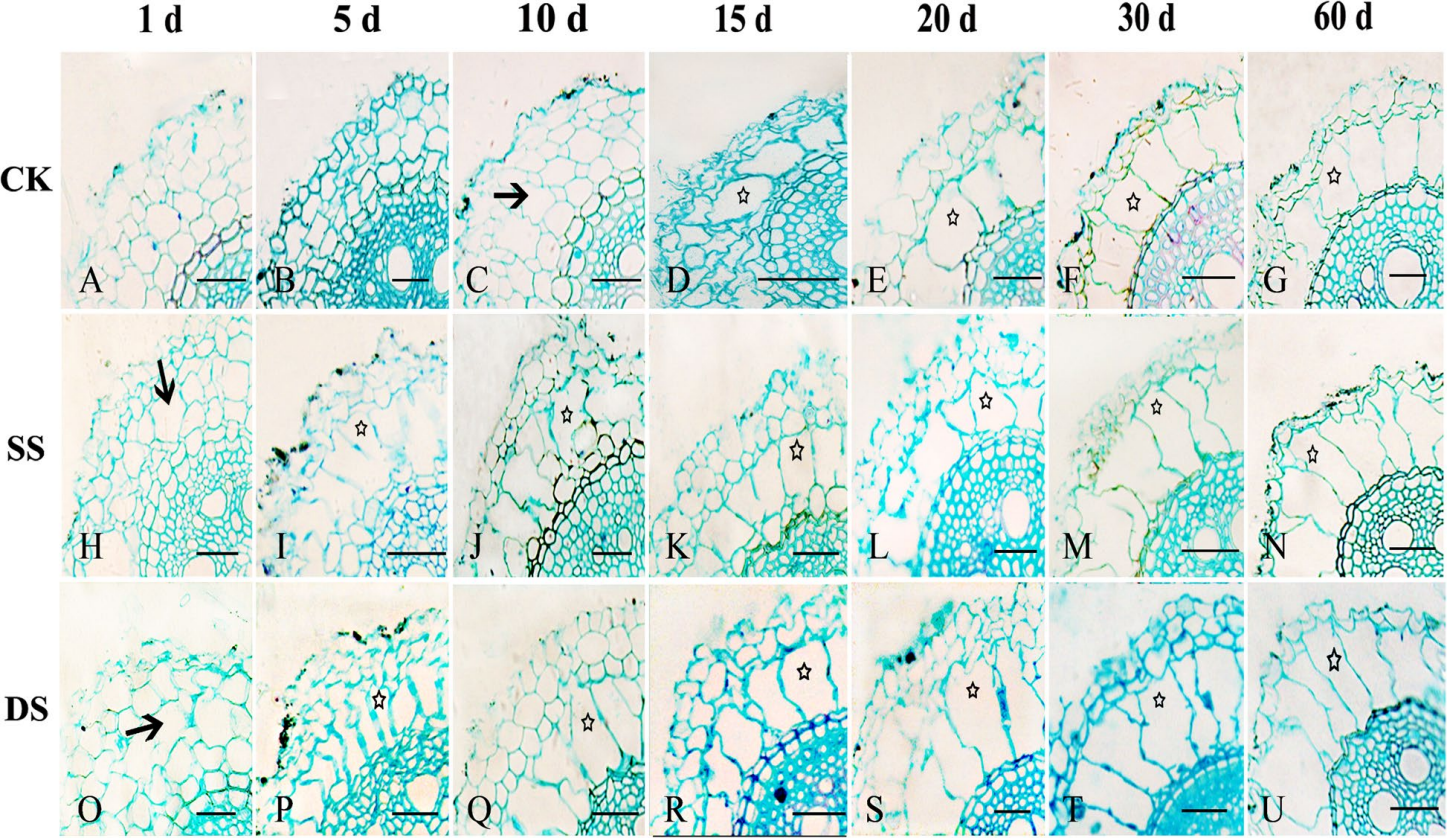
Aerenchyma of bermudagrass root under control (CK), shallow submergence (SS), and deep submergence (DS) treatment. The arrow indicates cavity, and the star indicates aerenchyma. Scale bar = 50 μm

We observed the transverse section of mature bermudagrass root (about 1 cm from root base) under different water treatments, and found that the root cavity rate showed a significant increase in three treatments during the entire experimental period. Bermudagrass root formed aerenchyma even in an aerobic environment. In CK, cortical cell growth led to an increase in intercellular space after 1 day, and began to shrink and invaginate after 5 days (Fig. 4A, B). Cortical cells formed cavities at 10 days post treatment, with root cavity rate being about 4% (Fig. 4C). Cortical cells then further developed aerenchyma at 15 days post treatment, with the root cavity rate being about 22% (Fig. 4D). Since then, the root cavity rate continuously increased, until reaching its maximum of 36% in CK at 60 days post treatment (Fig. 4G). Moreover, in SS and DS, the cortical cell of bermudagrass root formed cavities after 1 day, with the root cavity rate being about 6% and 3%, respectively (Fig. 4H, O). Cortical cells further developed aerenchyma after 5 days, with the root cavity rate reaching 24% and 21% in SS and DS, respectively (Fig. 4I, P). The root cavity rate of bermudagrass increased to its maximum about 45% and 44% in SS and DS at 60 days post treatment, respectively (Fig. 4N, U).

**Fig. 5:**
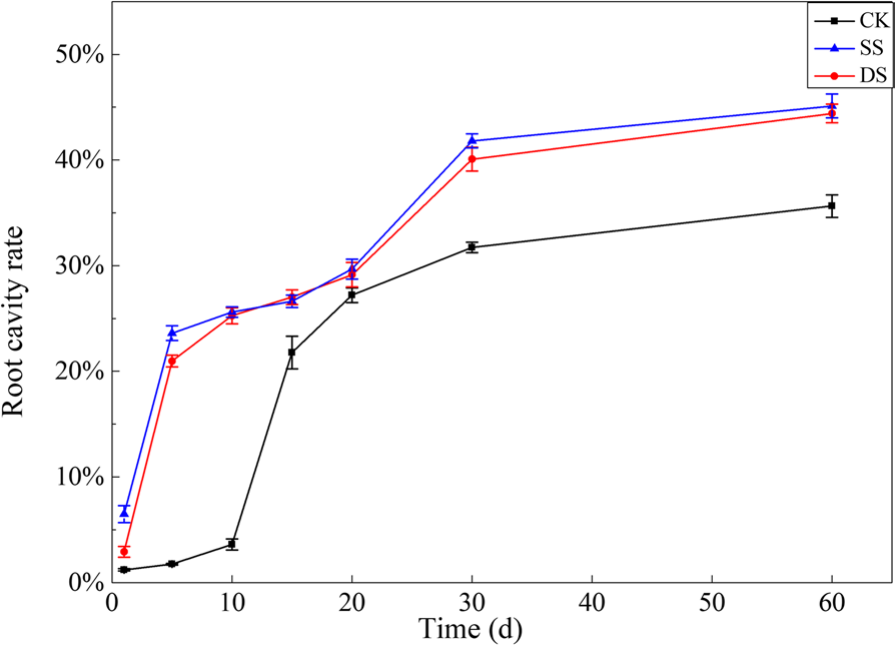
Root cavity rate of bermudagrass under control (CK), shallow submergence (SS), and deep submergence (DS) treatment. Values are mean ± standard error (SE) (*n* = 5)

The submergence stress significantly accelerated the cavity formation in bermudagrass (*P* < 0.001). At 60 days post treatment, the root cavity rate of SS and DS were 1.27 and 1.24 times that of CK, respectively (Fig. 5). Interestingly, at the end of the experiment, there was no significant difference in the root cavity rate between SS and DS (*P* > 0.05).

### Changes of cell microstructure during the aerenchyma formation

**Fig. 6:**
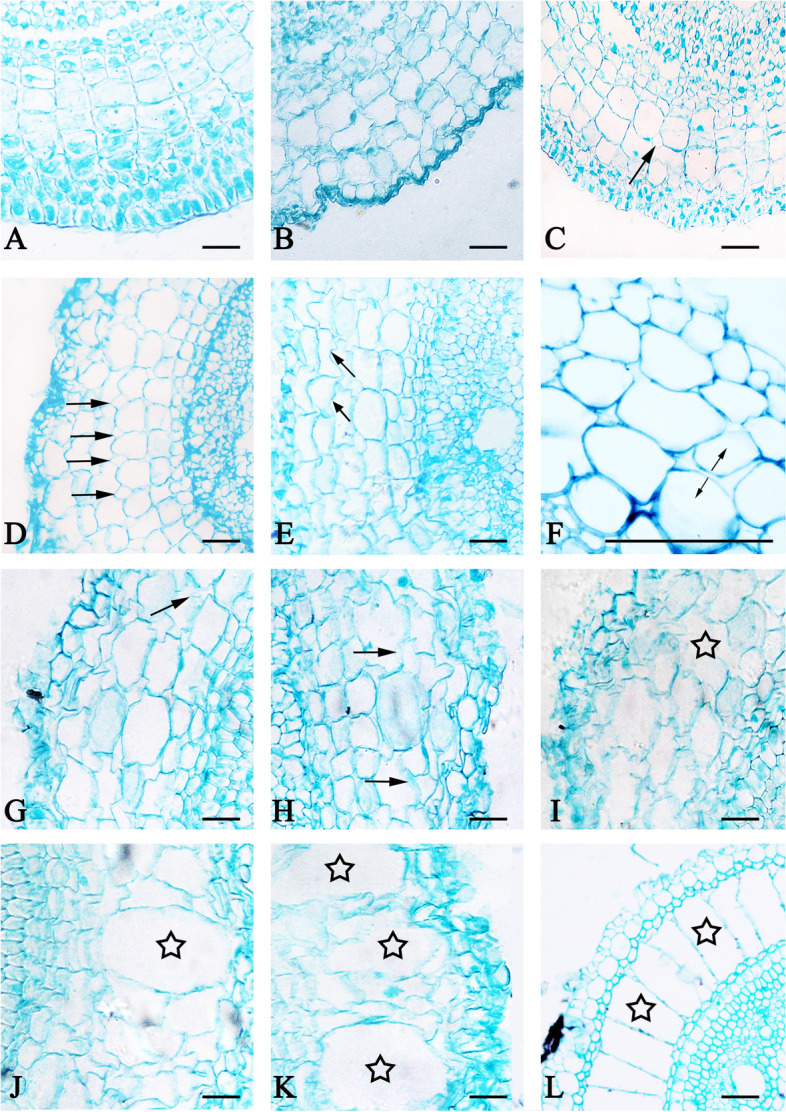
Microstructural changes of aerenchyma formation in the root cortex of bermudagrass. Pre-cavity phase (**A-B**): (**A**) the root was initially differentiated; (**B**) the cortical cells were closely arranged. Cavity formation phase (**C**-**D**): (**C**) intercellular space (arrow) was formed in the cortex; (**D**) intercellular space (arrow) was expanded to be maximal. Cavity expansion phase (**E**-**I**): (**E**) a few local cells showed invagination (arrow); (**F**) the adjacent cells further contracted (arrow); (**G**) cortical cells began to be autolyzed (arrow); (**H**) most cells in the cortex were autolyzed (arrow); (**I**) irregularly shaped cavities (star) were formed. Mature cavity phase (**J**-**L**): (**J**) developed aeration cavities (star) were formed; (**K**) more developed aeration cavities (star) were formed; (**L**) well-developed aerenchyma (star) was formed throughout the cortex. Arrows indicated cell gap, cell shrinkage direction, and dissolved cells, while asterisks indicated ventilation cavity and aerenchyma. Scale bar = 50 μm

The aerenchyma of bermudagrass root was a specialized form in cortical parenchyma cells. The inner and outer cortical structures remained intact and did not participate in the aerenchyma formation. The bermudagrass root was serially sectioned to observe microstructure changes of aerenchyma formation in cortical parenchyma tissue, as shown in Fig. 6. The processes of aerenchyma formation were divided into four phases: pre-cavity phase, cavity formation phase, cavity expansion phase, and mature cavity phase.

#### Phase 1: pre-cavity

At a distance of 0.1 cm from root tip, the root tip was in the primary meristem stage, comprising the cells of ground meristem with thick cytoplasm, large nucleus and many small vacuoles, and no obvious intercellular space (Fig. 6A). At a distance of 0.15 cm from the root tip, the number of fundamental meristem cell layers significantly increased, and cell vacuolation was obvious; and then, the cortical tissue was differentiated and formed, the cells were arranged closely relative to each other (Fig. 6B).

#### Phase 2: cavity formation

Accompanying the division and differentiation of the cortical cells, at a distance of 0.2 cm from root tip, intercellular space was formed due to the increasing cell volume, and the intercellular space size and number significantly increased with the cell growth (Fig. 6C). At a distance of 0.35 cm from the root tip, the growth of cortical cells stopped, the cell size and intercellular space reached a stable period, and the intercellular space enlarged to the maximum (Fig. 6D).

#### Phase 3: cavity expansion

At a distance of 0.5 cm from the root tip, certain cortical cells began to shrink, and a few local cells showed invagination (Fig. 6E); further contraction of cortical cells resulted in adjacent cells separating to form a narrow cavity (Fig. 6F); cortical cells began to autolyze and the cavities between cells gradually increased (Fig. 6G), and then most cells in cortical cells underwent autolysis (Fig. 6H); the entire cortical structure became loose and there were many irregularly shaped cavities in the cortical cells (Fig. 6I).

#### Phase 4: mature cavity

At a distance of 0.8 cm from the root tip, the residual cell wall gradually approached to the superposition, eventually forming aeration cavities in the radial direction (Fig. 6J), and then the number of developed aeration cavities in cortical cells increased (Fig. 6K). At a distance of 1.5 cm from the root tip, cortical cells of root formed developed-aerenchyma and showed a distinct radial tendency. The residual aerenchyma wall connected from the stele to the mechanical organization of the outer layer (Fig. 6L).

### Changes of cell ultrastructure during the aerenchyma formation

In order to further study the cytological changes of different developmental phases in aerenchyma formation, the transmission electron microscopy was used to observe the ultrastructural characteristics of cells.

#### Phase 1: pre-cavity

**Fig. 7:**
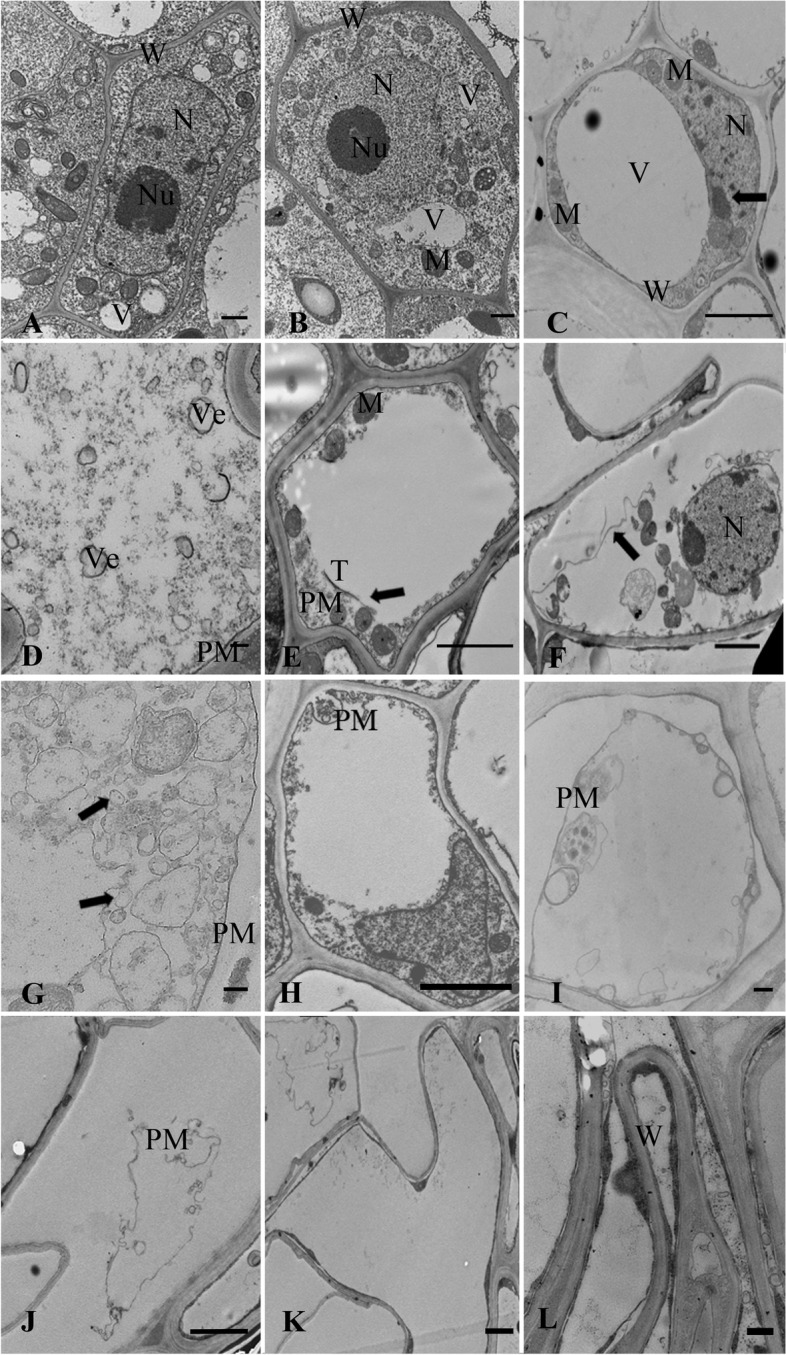
Ultrastructural changes of aerenchyma formation in the root cortex of bermudagrass. Pre-cavity phase (**A**-**B**): regular cell morphology, dense protoplasm and rich organelles. Cavity formation phase (**C**-**E**): (**C**) arisen central vacuole, chromatin agglutinated and marginalized (arrow); (**D**) a large number of vesicles; (**E**) broken vacuole membrane (arrow). Cavity expansion phase (**F**-**J**): (**F**-**G**) broken down plasma membrane (arrow) and appeared vesicles (arrow); (**H**-**J**) further degraded protoplasts and contents. Mature cavity phase (**K**-**L**): (**K**) completely degraded organelles; (**L**) deformed cell wall and formed aerenchyma cavity. W: cell wall, N: cell nucleus, Nu: nucleolus, PM: plasma membrane, V: vacuole, Ve: vesicle, T: tonoplast, M: mitochondria. Scale bars: A, B, D, G, I, L = 100 nm; C, E, F, H, J, K = 2 μm

At a distance of 0.1 cm from the root tip, cells were in the vigorous active period, which had the characteristics of regular cell morphology, dense protoplasm, large and round nucleus, smooth nuclear membrane, and conspicuous nucleolus. Several small vacuoles and organelles (i.e. plastid, mitochondria) in the protoplasts were also observed (Fig. 7A, B).

#### Phase 2: cavity formation

At a distance of 0.2 cm from root tip, with the development of cells, the degree of vacuolization was further enhanced and the volume of the vacuoles was enlarged. Finally, the large central vacuole being formed occupied most of the cell’s volume, and organelles such as mitochondria moved closer to the cell wall. The chromatin in the cell nucleus began to agglutinate and was marginalized (Fig. 7C). Afterwards, a large number of vesicles appeared in the cytoplasm and then the vacuole membrane was degraded and broken down (Fig. 7D, E).

#### Phase 3: cavity expansion

At a distance of 0.5 cm from root tip, the plasma membrane was broken down, the cytoplasm continued to degrade, and vesicles began to appear in the protoplasts (Fig. 7F, G). With the further degradation of protoplasts, most organelles degraded (Fig. 7H). Subsequently, the plasma membrane was further invaginated and lost its integrity, contents was further degraded, and an incompletely degraded membrane appeared (Fig. 7I, J).

#### Phase 4: mature cavity

At a distance of 0.8 cm from the root tip, the protoplasts entered the final stage of degradation, and all organelles were completely degraded (Fig. 7K). The cell wall was deformed and gradually degraded, gathering together to form a large aerenchyma cavity (Fig. 7L).

## Discussion

### Impact of submergence on biomass

Biomass changes are the most comprehensive adaptation of plants in response to the external stress environment [35]. In the face of varying degrees of submergence stress, plants have evolved various adaptive mechanisms to survive an aquatic environment [27]. They often use different biomass allocation strategies to cope with longer submergence [36].

In this study, the biomass increased and RSR decreased in CK with time. This is because the light, oxygen and water were sufficient, so the bermudagrass could grow normally and accumulate photosynthetic products. However, the transfer from shoot to the root storage is vital for the survival and recovery of bermudagrass under submergence stress. The change in both biomass and RSR in SS is due to the fact that in the face of a sudden submergence, bermudagrass consumed part of the energy (mainly losing part of the shoot biomass) for rapid physiological and metabolic response. Subsequently, the overgrowth of plants on the water surface increased the contact area with air to increase oxygen availability, while forming a large number of aerenchyma in response to submergence stress (Fig. 4) [37]. The biomass of bermudagrass was significantly reduced at 60 days post treatment in DS, which indicated that bermudagrass could improve the adaptability to the complete submergence environment by inhibiting growth and losing part of the biomass [38]. The results of CK and DS were consistent with previous studies [38, 39].

### Influence of submergence on root growth

There is a close relationship between root morphology and root biomass. In this study, the total root length, root volume and root surface area were consistent with changes in root biomass in all treatments. It is widely accepted that root morphology may play an important role in the response of roots to submergence [28]. In general, the length of root system depends on the root tip portion to obtain the oxygen needed to maintain breath [40]; and the increase in surface area can enhance root ability to absorb nutrients [41]. Because the rhizosphere oxygen was sufficient in CK, the root system could produce a large amount of ATP through aerobic respiration. The root system could continuously grow and absorb more nutrients, showing that total root length, root volume and root surface area continued to increase with time. However, the lack of oxygen in the rhizosphere in SS led to a decrease in ATP, which in turn inhibited the elongation of roots [42, 43]. At 15 days post treatment, bermudagrass responded to the submergence environment through a series of acclimations. For example, the formation of aerenchyma transported more oxygen to the roots to maintain the root’s energy consumption. The lack of oxygen caused the ATP synthesis to be blocked and mineral absorption to decrease, resulting in relatively weak physiological activities and root elongation growth in DS [44].

The surface area per unit volume of root could reflect the thickness of root, to a certain extent, and the increase of surface area per unit volume of root could increase the absorption capacity for nutrients [45]. In general, further development of aerenchyma is characterized by an increase in root diameter [46]. The larger the root diameter, the larger the area of the cortex where can be used to form aerenchyma, and thus the more developed aerenchyma. Therefore, the larger root diameter can reduce the diffusion resistance of the gas inside. In our study, roots can absorb more nutrients by increasing the surface area per unit volume of root in CK with sufficient oxygen. However, the root oxygen is significantly deficient in submergence conditions, and hence roots absorb more oxygen by increasing the root diameter (lower surface area per unit volume of root), thereby improving the oxygen content in the roots. The change of root diameter in our current study was consistent with that of previous studies [39]. Aerenchyma could reduce the diffusion resistance of oxygen in the internal space and transport oxygen to the roots to meet bermudagrass’ growth needs. Thus, the formation of aerenchyma played a crucial role in the transport of oxygen in the submergence environment. In the present study, the roots were still white at 60 days post treatment, indicating that the activity of roots was still vigorous and there was no death of the roots, further suggesting that the influence of submergence on bermudagrass was very limited, and bermudagrass roots played an important role in submergence tolerance.

### Influence of submergence on aerenchyma formation

The formation of aerenchyma is closely related to the external environmental conditions of the root system. Under different environmental conditions, the formation of aerenchyma varies. In this study, developed aerenchyma was formed in bermudagrass root in CK at 15 days post treatment, while developed aerenchyma was formed in bermudagrass root in the SS and DS after 5 days, which was similar to previous studies in roots of rice (*Ory sativa*) and maize (*Zea nicaraguensis*) [47, 48]. The former and the latter were defined as constitutive aerenchyma and induced aerenchyma, respectively. The formation of constitutive aerenchyma can effectively transport oxygen to the roots, allowing the bermudagrass to continue to breathe in a sudden submergence environment, while the death of the root cortical cells caused by aerenchyma formation reduced the root energy consumption and nutrient requirements (per unit root length). In addition, formation of constitutive aerenchyma provided oxygen for ethylene production, resulting in ethylene-induced aerenchyma formation [28]. The formation of constitutive aerenchyma was more beneficial to bermudagrass as it could help bermudagrass quickly acclimate to submergence conditions through improvement of the submergence tolerance [48–50].

Submergence accelerated the aerenchyma formation of bermudagrass root and increased the root cavity rate of aerenchyma. Such similar results were also obtained in previous studies of wheat (*Triticum aestivum*) [24, 51], rice [47], and maize [52]. Accelerated formation of aerenchyma can improve the efficiency of oxygen transport inside. This process is of great significance for the survival of bermudagrass under submergence conditions. It is easily understood that the formation of aerenchyma in stems and roots helps transport oxygen from the environment to anoxic roots through low-resistance channels, and can also transport CO_2_, methane and ethylene from soil to the atmosphere [19]. However, different depth and duration of submergence imposes distinct environmental constraints on different organ [53]. Therefore, an integrated study of the changes of aerenchyma in different tissues and organs (roots, stems and leaves) under different submergence conditions should be considered in the follow-up research, which is of great significance to facilitate deeply understanding the adaptive mechanisms of bermudagrass to flooding stress. Such an integrated study can provide references for breeding new flooding-tolerance bermudagrass varieties in the future.

### The manner and process of aerenchyma formation

From the microstructure and ultrastructure results of whole experiment, the pre-cavity phase of aerenchyma formation in bermudagrass root mainly had the microstructure characteristics of thick cytoplasm, large nucleus, and many small vacuoles. Likewise, we found the same results in ultrastructure, such as dense protoplasm, large and round nucleus, and several small vacuoles. The microstructure of cavity formation phase included the enlargement of cell volume and then reached stable. Meanwhile, we observed that there were large central vacuole, agglutinated and marginalized chromatin, lots of vesicles, and degraded vacuole membrane. Moreover, the separation between cells formed a narrow cavity, and then cells gradually died to dissolve and form large cavity. At the same time, the results of plasma membrane broken down, and cytoplasm and contents degradation were also observed in the ultrastructure. Lastly, the residual cell wall gradually approached to the superposition, and eventually formed a developed “spoked wheel” aerenchyma in mature cavity phase. In the study of the aerenchyma formation in *Triticum aestivum*, the phenomenon of intercellular space enlargement, cortical cell contraction and “spoked wheel” aerenchyma were found [54]. Besides, the former study also documented that the aerenchyma in *Triticum aestivum* was formed by both schizogenous and lysigenous process, with schizogenesis occurring in the front and lysogenesis occurring in the back and gradually dominating [54]. Furthermore, it was reported that the formation of aerenchyma in *Trapa pseudoincisa* was the result of cell separation along the radial wall and cell death, which was known as schizo-lysigenous aerenchyma [27]. In the present study, the cell separation and PCD typical biomarkers were also observed in the aerenchyma of bermudagrass root. As the afore-mentioned microstructure and ultrastructure results in our study were consistent with the previous ones [27, 54], the formed aerenchyma in bermudagrass root can thus be considered as schizo-lysigenous aerenchyma as well.

## Conclusion

In conclusion, deep submergence inhibited the accumulation of biomass and root growth of bermudagrass, and shallow submergence only had a limited influence in this regard in earlier experiment period. But, bermudagrass responded to submergence conditions by changing its root morphology (increasing root diameter) and anatomy (increasing aerenchyma formation) to maintain root system functioning. Meanwhile, the results of microstructure and ultrastructure showed that PCD occurred in the process of constructive aerenchyma formation in bermudagrass root to form schizo-lysigenous aerenchyma. This study mainly elucidated the response strategies of bermudagrass roots to different submergence treatments and the manner of aerenchyma formation under non-flooding condition. To understand bermudagrass aerenchyma formation process completely, it is of necessity to continuously study the manner of aerenchyma formation under flooding conditions. Moreover, further investigation of the regulation mechanism of signal molecules in the PCD process of bermudagrass aerenchyma formation under different water regimes should also be pursued in future experiments.

## Materials and methods

### Plant materials and growing conditions

The experiment materials comprised of bermudagrass and purple soil which were collected from the artificial revegetation site of Ruxi river hydro-fluctuation belt in the TGR, Shibao town, Zhong county, Chongqing municipality of China (107°32'—108°14' E, 30°03 '—30°35' N). On 12 April 2018, we planted bermudagrass cutting (10 cm long stolon with 2 nodes for each cutting) in the container (with the size of outer diameter 8.8 cm × inner diameter 8 cm × bottom diameter 6 cm × height 8 cm). There were a total of 331 plants for the experiment. All of the cuttings were put under an experimental booth of the Key Laboratory of Eco-environments in the Three Gorges Reservoir Region (Ministry of Education, 249 m above sea level), and watered normally (with soil moisture content being 60—63% of soil field water capacity) during the cultivation process. The soil field moisture content was determined by weighing, and soil moisture content of container was determined by Soil Three-parameter Meter (WET, US). This research was conducted in the authorized laboratory and there was no need of specific permission for sampling plants and soils.

### Experimental design

All the plants were acclimated under an experimental booth (average temperature about 20 °C, and average relative air humidity about 74%) for 10 days, and then treatments began on 22 April 2018, when the bermudagrass roots were 3–5 cm long. Among the total 331 cuttings, 16 cuttings were randomly selected for a baseline measurement (*n* = 10) and aerenchyma formation process observation (*n* = 6). Meanwhile, the other 315 cutting plants were randomly placed into 9 plastic buckets (with the bucket size of bottom long 95 cm × bottom wide 75 cm × high 66 cm, 35 containers in each plastic bucket). They were then treated with three different water conditions (three replicate buckets under one water gradient), including control (CK)—normal water supply, with soil moisture content being 60%—63% of soil field water capacity; shallow submergence (SS)—submergence 5 cm above the soil surface; and deep submergence (DS)—submergence over the top of the plant 50 cm. The submergence depth of the SS and DS was always 5 cm and 50 cm throughout whole period. Samples were taken after 1, 5, 10, 15, 20, 30, and 60 d at 7:00 am on the day of sampling. At each sampling time, 5 plants of bermudagrass were randomly selected from each plastic bucket for destructive sampling under each treatment. Thus, 15 plants were taken from each treatment at each sampling time for the determination of plant biomass and root morphology (*n* = 10) and aerenchyma microstructure and root cavity rate (*n* = 5).

### Assay of plant biomass and root morphology

At different treatments and different sampling times, 10 plants were randomly harvested and washed under running water, and then divided each plant into shoots and roots. Total root length, root surface area, root volume, and average root diameter of each individual plant were measured by a root analysis system (WinRhizo Pro. 2004c, Regent, Canada) [55]. Finally, shoots and roots were dried at 80℃ to constant weight and gravimetrically measured [56].

### Assay of aerenchyma microstructure and root cavity rate

Assay of aerenchyma microstructure at a distance of 1 cm from root bases and the root cavity rate under different water conditions were conducted. Five root segments of the portion between 0.9–1.1 cm starting from the root base were randomly selected under different treatments and different sampling times and fixed in formalin-aceto-alcohol (FAA). The root segments were dehydrated with gradient alcohol (70%, 85%, 95%, 100%, 100%), xylene transparent, dip wax, and then embedded. The embedded material was sectioned with a rotary paraffin slicer (FINESSE 325, Thermo, US), sliced to 10 μm thickness, then dewaxed, gradient alcohol rehydrated, and stained with safranin and fast green. Finally, we observed the transverse section at a distance of 1 cm from root bases and photographed under a microscope (ECLIPSE 80i, Nikon, Japan), and then determined the root cavity rate by IPP (Image-Pro Plus 6.0) software [24, 54]. Root cavity rate refers to the area of the intercellular spaces (including normal cell gap, schizogenous space, and lysigenous space) on the transverse section of the root as a percentage of the root.

### Observation of aerenchyma formation process

For the microstructure observation of aerenchyma formation process, three root segments (i.e. 2 cm length root tips) were randomly taken from bermudagrass in CK and were fixed in the FAA. Then, the root segments were serially sectioned by conventional paraffin section (see above). Finally, the microstructure was observed and photographed under a microscope (ECLIPSE 80i, Nikon, Japan) [24]. Meanwhile, to observe the ultrastructure of aerenchyma formation process, three root segments (i.e. 2 cm length root tips) were also taken from bermudagrass in CK and were fixed in 0.1 M phosphate buffer saline (PBS) buffer (pH 7.0) containing 2.5% glutaraldehyde at 4℃ overnight. The root segments were rinsed in 0.1 M PBS buffer (pH 7.0) three 30-min, and then post-fixed in 1% osmium tetroxide overnight at 4℃ in 0.1 M PBS buffer (pH 7.0). Afterward, the root segments were dehydrated with gradient alcohol, final change in 1,2-epoxypropane, and embedding. 0.10 μm thickness sections were obtained using ultramicrotome (Leica EM UC6), and then were mounted on copper grids and stained with uranyl acetate and lead citrate. Finally, they were observed for ultrastructure and photographed under an H-7650 transmission electron microscope (Hitachi) [27].

### Statistical analysis

Data in the experiment were statistically analyzed using SPSS 22.0 software. Two-way ANOVA was used to assess the influence of submergence treatment, sampling time, and their interactions on root morphological characteristics and biomass of bermudagrass. Tukey test was then used to determine significant differences at the 0.05 level between different treatments. Figures were conducted by Origin Pro 9.0 (Origin lab Corporation) and composed by Adobe Photoshop (Adobe Systems).

## Data Availability

All data generated or analyzed during this study are included in this published article.

## Abbreviations

ATP: Adenosine Triphosphate
CK: Control
DS: Deep Submergence
FAA: Formalin-Aceto-Alcohol
PBS: Phosphate Buffer Saline
PCD: Programmed Cell Death
ROS: Reactive Oxygen Species
RSR: Ratio of Shoot to Root in biomass
SS: Shallow Submergence
TGD: Three Gorges Dam
TGR: Three Gorges Reservoir
